# Rapid and Sensitive Isothermal Detection of Nucleic-acid Sequence by Multiple Cross Displacement Amplification

**DOI:** 10.1038/srep11902

**Published:** 2015-07-08

**Authors:** Yi Wang, Yan Wang, Ai-Jing Ma, Dong-Xun Li, Li-Juan Luo, Dong-Xin Liu, Dong Jin, Kai Liu, Chang-Yun Ye

**Affiliations:** 1 State Key Laboratory for Infectious Disease Prevention and Control, National Institute for Communicable Disease Control and Prevention, Chinese Center for Disease Control and Prevention, Changbai Road 155, Changping, Beijing 102206, PR China; Collaborative Innovation Center for Diagnosis and Treatment of Infectious Diseases, Hangzhou, PR China; 2 Department of Microbiology, Guiyang Medical University, Guiyang, Guizhou 550004, PR China; 3 Pathogenic Biology Institute, University of South China, Hengyang, Hunan 421000, PR China

## Abstract

We have devised a novel amplification strategy based on isothermal strand-displacement polymerization reaction, which was termed multiple cross displacement amplification (MCDA). The approach employed a set of ten specially designed primers spanning ten distinct regions of target sequence and was preceded at a constant temperature (61–65 °C). At the assay temperature, the double-stranded DNAs were at dynamic reaction environment of primer-template hybrid, thus the high concentration of primers annealed to the template strands without a denaturing step to initiate the synthesis. For the subsequent isothermal amplification step, a series of primer binding and extension events yielded several single-stranded DNAs and single-stranded single stem-loop DNA structures. Then, these DNA products enabled the strand-displacement reaction to enter into the exponential amplification. Three mainstream methods, including colorimetric indicators, agarose gel electrophoresis and real-time turbidity, were selected for monitoring the MCDA reaction. Moreover, the practical application of the MCDA assay was successfully evaluated by detecting the target pathogen nucleic acid in pork samples, which offered advantages on quick results, modest equipment requirements, easiness in operation, and high specificity and sensitivity. Here we expounded the basic MCDA mechanism and also provided details on an alternative (Single-MCDA assay, S-MCDA) to MCDA technique.

**N**ucleic acid amplification is a valuable molecular tool in modern biology and medicine, and is widely applied in basic research, clinical diagnosis, forensic science, epidemiology, agriculture and many other fields^1^. Among the many methods established for the amplification and analysis of nucleic acids, polymerase chain reaction (PCR) provides a general protocol for the rapid detection, isolation, and measurement of DNA sequences through their specific amplification^2^. However, PCR relies upon complicated thermal cycling steps for successful DNA amplification, and the resultant instrumental restraint has been limiting its more flexible and wider applications^3^. Herein, the development of alternative assays for the simple, rapid and sensitive detection of DNA is in continuous demand.

Accordingly, a wide variety of isothermal amplification approaches (e.g., rolling circle amplification, strand displacement amplification, helicase-dependent amplification, cross-priming amplification, loop-mediated isothermal amplification *et al.*) have been reported, which obviate the use of a thermocycling apparatus, can be real-timely detected and favor point-of-care (POC) diagnosis^4^. However, these assays are still relatively complex protocols requiring rigorous optimization, the use of multiple enzymes (two or more) and/or special reagents, with no considerable improvement in their operativity, sensitivity and specificity^5^. In order to achieve amplification, an enzyme based techniques for promoting nicking and strand displacement, or a high temperature denaturation step is particularly required by these approaches. The methodology presented in this study, multiple cross displacement amplification (MCDA), overcomes the technical difficulties posed by current isothermal amplification techniques. The novel approach would be useful that combines cost-efficiency (e.g., using a single enzyme), robustness (e.g., completing reaction at a constant temperature), high specificity (e.g., recognizing multiple sequences of the target) and sensitivity (e.g., amplifying trace amounts of DNA) for rapid molecular analysis of clinical, environment or biological samples in both basic and applied research.

In MCDA, the isothermal amplification of specific nucleic-acid sequences is achieved by employing a set of 10 primers spanning 10 distinct regions of target fragment, which are designated as cross primers (CP1 and CP2), displacement primers (F1 and F2) and amplification primers (D1, C1, R1, D2, C2 and R2) (Fig. 1). The MCDA assay, as a novel isothermal nucleic acid amplification strategy, utilizes only a polymerase with strand displace activity to amplify the target. In the MCDA assay, cross primer CP1 initiates MCDA reaction at the P1s site of the target, which will be displaced by upstream synthesis from the F1 primer (Step 1). After 3 amplification primers (D1, C1 and R1), cross primer CP2 and displacement primer F2 anneal to the newly synthesized strand and the polymerase extends in tandem creating 4 different products (Step 2). The D1 product can be extend by amplification primer C1 and cross primer CP1 to form a CP1/D1 product (Step 3). The C1 strand is complementary at its 3' and 5'ends by constraint of the introduction of the C1s sequence at the 3'end of the top strand and the C1 sequence at the 5'end. The newly displaced strand forms an intramolecular stem-loop structure which is hybridized and extended by the cross primer CP1, generating a CP1/C1 product (Step 4). The two strands from the CP1/C1 product also yield stem-loop structures which can be re-annealed and stabilized by the base pair double helix formed between C1 and C1s. Then, these DNAs can be used as templates for further extension by amplification primers D1, C1 and cross primer CP1, forming CP1/D1 and CP1/C1 products (Cycle 1). For the R1 strand, the amplification process is similar to C1 strand, yielding R1/R1s, CP1/R1, CP1/D1 and CP1/C1 products (Cycle 2). The CP2 is the only other cross primer of the MCDA detection system and will be displaced when the DNA polymerase extends the upstream displacement primer F2 (Step 2). At the 3’end, the CP2 product can hybridize to this strand, thus a new strand with stem-loop structure is produced (Step 6). Moreover, the amplification primers D2, C2 and R2 anneal to the new CP2 strand, and the amplification process is similar to primers D1, C1 and R1 (Step 7). For the subsequent amplification, these new strands with stem-loop structure enter the exponential amplification and strands with several inverted repeats of the target DNA can be generated by strand displacement and repeated extension (Step 8, 9, 10 and 11). Here, we illustrate the optimization of the basic MCDA reaction and demonstrate an application of the MCDA assay.

## Results

### The primer design for MCDA assay

In order to demonstrate the mechanism of MCDA, the *hlyA* gene (GenBank GeneID: 223702383), which was specific for *L. monocytogenes* species, was chosen as a model DNA^6^. A set of ten primers, including 2 cross primers (CP1 and CP2), 2 displacement primers (F1 and F2) and 6 amplification primers (C1, D1, R1, C2, D2 and R2), which recognized 10 distinct regions more than 200 base pairs on *hlyA* gene, were designed by using PrimerExplorer V4 (Eiken Chemical) and primer software PRIMER PREMIER 5.0 according to the mechanism of MCDA. The specificity of MCDA primers was determined using the NCBI BLAST (Basic Local Alignment Search Tool), and no exact match against *L. innocua*, *L. seeligeri*, *E. coli* and *S. enterica*. The details of target sequence, primer design, primer locations and sequences were shown in Fig. 2 and Table 1.

### Confirmation and detection of MCDA products

To confirm the correct amplification of MCDA, amplification systems were performed in the presence or absence of genomic DNA templates at 63°C. Two determination methods, including colorimetric indicators (such as FD reagent) and agarose gel electrophoresis, were selected for monitoring the MCDA reaction^7^.

The FD reagent can be added directly during the MCDA reaction mixture preparation, and indicates the result of the MCDA reaction by the color change. Thus, the positive results can be detected from the change of color by the naked eye. In the MCDA tubes, the color change of positive reactions from light gray to green was directly observed by naked eye, while the negative control tube remained light gray (Fig. 3A).

**Figure 1:**
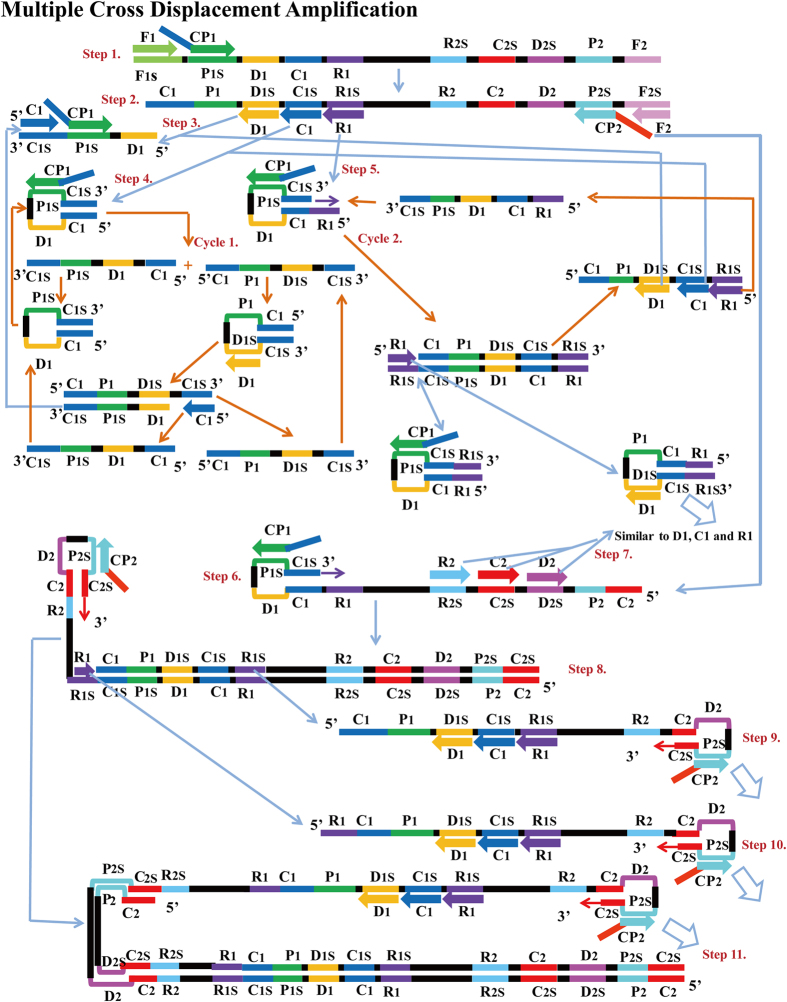
The principle of multiple cross displacement amplification. The schematic showing the mechanism of the novel MCDA assay.

The final products of the MCDA reaction are mixtures of various short segments of DNAs (Fig. 1 structures 3, 4, 5 and 7) and stem-loop DNAs with different stem lengths formed by annealing between alternatively inverted repeats of the target DNA in the same sequence (Fig. 1 structures 6, 8, 9, 10 and 11). Therefore, electrophoretic analysis of the MCDA amplified products, the characteristic ladder-liker pattern bands on agarose gel electrophoresis should be yielded in positive amplification but not in the negative control. As expected, the positive results were observed many bands of different sizes in a ladder-like pattern on ethidium bromide-stained 2.5% agarose gel electrophoresis (Fig. 3B). These results suggested that the novel MCDA technique could correctly amplify DNA under isothermal conditions.

### Validation of the reliability of MCDA by sequencing

To further confirm the reliability and specificity of MCDA assay, a MCDA-PCR-sequencing strategy was developed to analyze the MCDA products. The sequences of PCR amplicons, which amplified from MCDA products, were 98% match with the expected sequences (Fig. 4). The sequencing data showed that the correct amplification of MCDA was further validated.

### The optimal temperature of MCDA assay

For an isothermal amplification assay, the key is the establishment of a dynamic reaction environment at a constant temperature that balances binding of the primer to the template strand with elongation activity of the polymerase. Herein, the MCDA reactions were tested at different temperatures (60–67 °C) with the appropriate primers and the reference strain EGD-e was selected as positive control to determine the optimal temperature at the level of 250 pg genomic DNA per reaction. The results were analyzed by real-time measurement of turbidity and a typical kinetics graph was shown in Fig. 5. The positive products were also visualized as a ladder-like banding pattern on agarose gel electrophoresis (Fig. 6). The reaction temperatures of 61–65 °C were recommended as the standard temperatures for the MCDA assay and the temperature of 63 °C was selected for the rest of MCDA amplification conducted in this study.

### Analytical sensitivity of the MCDA assay

Sensitivity of MCDA reaction on *L. monocytogenes* was examined by analyzing the products yielded from the serial dilutions (2.5 ng, 250 pg, 25 pg, 2.5 pg, 250 fg, 125 fg, 62.5 fg and 31.25 fg per microliter) of the *L. monocytogenes* genomic DNA at least three times. As shown in Fig. 7A, the MCDA reactions were monitored by real-time turbidity detection; the decreasing amounts of DNA were listed from left to right and the LoD of MCDA assay was 62.5 fg DNA per reaction. The positive reactions by FD reagent were visualized as the change of color from light gray to green, and the positive results by 2.5% agarose gel electrophoresis were seen as a ladder-like pattern (Fig. 7B,C). The LoD of the two monitoring methods for MCDA reaction was 62.5 fg DNA per reaction, which was identical with turbidity detection. Moreover, the MCDA reactions only require 40-min incubation periods at LoD levels of genomic DNA (Fig. 7A).

The LoD of LAMP and CPA reactions on *L. monocytogenes* was 1 pg DNA/tube and 2 pg DNA/tube, respectively, whereas the LoD of MCDA assay was 62.5 fg DNA/tube (Fig. 8, Table 2). These results showed that the MCDA approach was 16-fold and 32-fold more sensitive than the LAMP and CPA assays for detecting *L. monocytogenes* genomic DNA, respectively. Moreover, the LAMP and CPA reactions required 52- and 63-min incubation periods at LoD levels of template DNA, respectively (Table 2).

### Specificity of the MCDA assay

To evaluate the specificity of the MCDA approach, the *L. monocytogenes* MCDA assay was carried out under the standard conditions described above with the genomic DNA extracted from the 40 non-*L. monocytogenes* and 50-*L. monocytogenes* strains (Table 3). The positive results by FD reagent were directly seen as the color change in all *L. monocytogenes* strains, but not in non-*L. monocytogenes* strains (Fig. 9A). As shown in Fig. 9B, the typical ladder-like pattern on 2.5% agarose gel electrophoresis was produced in all *L. monocytogenes* strains, whereas the non-*L. monocytogenes* strains did not show the characteristic pattern. These results indicated that the *L. monocytogenes* MCDA assay was highly specific to the pathogen identification.

### Practical application of MCDA to *L. monocytogenes* detection in pork samples

For the purpose of demonstrating the applicability of the MCDA assay as a nucleic acid analysis tool, the *L. monocytogenes*-MCDA method was applied to detect the foodborne pathogen in pork samples. Fifty-five pork samples were analyzed using PCR, MCDA and traditional culture-biotechnical assays. The results were summarized in Table 4. Of a total of 55 pork samples, 14 (25.5%) were *L. monocytogenes* positive by the novel MCDA assay which was in accordance with the results by culture-biotechnical method, and 8 (14.5%) for the conventional PCR method. These results suggested that the proposed methodology was a good alternative to conventional culture-biotechnical technology and could provide a potential for nucleic acid analysis.

### Single-MCDA assay

Here, we also reported an alternative to MCDA technology, which was termed single-MCDA (S-MCDA). In the S-MCDA reaction system, 3 amplification primers (R2, C2 and D2; or R1, C1 and D1) were removed, which reduced the requirement for conservation and length of the target sequences. Thus, the MCDA system was separated into two single-MCDA reactions and the primers set of S_1_-MCDA reaction system consists of CP1, CP2, F1, F2, R1, C1 and D1, S_2_-MCDA for CP1, CP2, F1, F2, R2, C2 and D2.

In order to demonstrate the correct amplification of S-MCDA, two monitoring methods, including FD reagent and agarose gel electrophoresis, were applied to determine the S-MCDA reactions. A positive color (green) was visualized in the presence of *L. monocytogenes* genomic DNA, a negative color (light gray) for the control tube (Fig. 10A,C). The positive DNA products were observed as a ladder-like banding pattern on 2.5% agarose gel electrophoresis (Fig. 10B,D).

The sensitivity of S_1_-MCDA and S_2_-MCDA reactions was also assessed by analyzing the products generated from the serial dilutions (2.5 ng, 250 pg, 25 pg, 2.5 pg, 250 fg, 125 fg, 62.5 fg and 31.25 fg per microliter) of the *L. monocytogenes* genomic DNA, and three replicates of each dilution were tested. The LoD of S_1_-MCDA and S_2_-MCDA was 125 fg DNA/reaction (Fig. 11). The positive results of S_1_-MCDA and S_2_-MCDA were produced in as short as 25 minutes and the two S-MCDA reactions required 50-min incubation periods at the LoD levels of DNA (Fig. 11A,B). The specificity of S_1_-MCDA and S_2_-MCDA reactions was determined using the genomic DNA extracted from 50 *L. monocytogenes* and 40 non-*L. monocytogenes* strains. The DNA of all *L. monocytogenes* was tested positive with the two sets of primers, whereas no amplification of the non-*L. monocytogenes* genomic DNA was observed (data not shown).

## Discussion

The adoption of nucleic acid amplification *in vitro* has resulted in powerful technologies that underpinned biological research and diagnostics. Because of the requirement for complicated apparatus, special reagents and multiple enzymes, these techniques were used as special tools available only to particular fields, facilities and laboratories^8^. However, these technologies are constantly evolving and the design of alternative methodology for simple, sensitive and specific detection of nucleic acids is continually emerging.

In this study, we presented the MCDA technology, as an isothermal amplification technique, which was carried out at a constant temperature (61–65 °C). Thus, MCDA did not require thermal denaturation of templates, and eliminated the use of the temperature-regulating equipment. Therefore, the MCDA can be as a potential tool for developing hand-held diagnostic devices, which can be used to detect pathogens and diagnose clinical disease in research on environment hygiene, point-of-care diagnosis, ‘on-site’ testing and more.

Achieving efficient genetic analysis with the MCDA assay relies not only on the performance of nucleic acids amplification, but is also dependent on the technique selected for monitoring the MCDA reaction^7^. The FD colorimetric indicator, a simple visual detection method for the results of the MCDA reaction, presents visual discrimination of the positive amplification within 40-min, alleviating the use of costly specialized equipment^9^. Moreover, the FD reagent allows for a single-step MCDA assay and the reaction tubes are not opened after the amplification, thus the risk of post- or cross-contamination is much lower^10^. Therefore, the MCDA technique combined with FD reagent should be useful in various fields, such as field diagnosis, point-of-care testing and more. Gel electrophoresis has been used as a mainstream method for detecting the amplicons of isothermal amplification approaches, even as a “gold standard” in many situations^11^,^12^. Hence, the technique was also employed to detect MCDA amplicons, and the positive reactions produced many bands of different sizes upon 2.5% agarose gel electrophoresis because the MCDA products consist of various short segments of DNAs and stem-loop DNAs with different stem lengths (Fig. 3B). Moreover, the results of the MCDA amplification were monitored in real-time with optical instruments (turbidimeters), and the detection results were reported in a typical kinetics graph (Figs 5,7 and 11). The real time turbidity method did not require special indicators, probes or auxiliary regents except for the turbidimeter. Likewise, the risk of amplicon contamination was also obviated.

In the MCDA assay, a set of ten primers with ten binding sites, which hybridizes correctly to target sequences, provides a high degree of specificity, and the detection specificity was verified in our study. The primers in the MCDA assay only amplified the genomic DNA from *L. monocytogenes* strains and did not amplify the templates from the non-*L. monocytogenens* strain and negative control (Fig. 9). The results demonstrated that the novel MCDA technique offered high selectivity for analyzing the target sequences.

The newly established MCDA assay detected as little as 62.5 fg DNA per reaction and was at least 160-fold more sensitive than that of PCR, which has been report for *L. monocytogenes* detection^13^,^14^,^15^. Due to reduce the need for sophisticated equipment, have short turnaround times and low running costs, the MCDA assay is more suitable than PCR for simple, sensitive and rapid detection in various fields. Comparing with other isothermal amplification approaches (such as LAMP, CPA), MCDA was 16- and 32-fold more sensitive than the LAMP and CPA assays, respectively, and the positive reactions were obtained in as short as 15 minutes (Table 2, Fig. 7A). Thus, our assay has advantages over other isothermal techniques, namely high sensitivity and quick results, and is a good alternative to these methods for detection of trace amounts of target sequences in various samples.

In order to determine the practical application of MCDA detection to *L. monocytogenes* in food samples, 55 pork samples were analyzed by PCR, conventional culture-biotechnical detection and MCDA methods. Six pork samples were positive for *L. monocytogenes* using the MCDA and culture-biotechnical assays, but negative by PCR. Thus, the MCDA offered higher analysis sensitivity for the practical application compared to PCR. The poor sensitivity of PCR may be due to the reasons that the presence of non-target DNA in samples and some inhibitors (such as serum, blood and food ingredients) specific to the PCR affected the reaction sensitivity, or the copy numbers of the target templates were lower than the limit of detection^16^,^17^,^18^. Thereby, MCDA method is more tolerant to PCR inhibitors in samples and does not appear to be affected by presence of non-target sequences. Moreover, our study verified 100% agreement in *L. monocytognenes*-specific detection using MCDA and culture-biotechnical methods, while the MCDA assay was less expensive and timesaving.

The MCDA was formulated and developed as an isothermal amplification with the requirement for a set of 10 primers, which recognized 10 distinct regions of target sequences. In this manifestation, the MCDA technology has been verified as a rapid, highly sensitive and specific analysis tool for a target nucleic acid sequence. The primer design for MCDA technique requires the selection of 10 separate binding sites, and the CP1 and CP2 primers, containing at 5' end of the sequence complementary to C1 and C2, presented the restrictions on their positioning respective to each other. As a sequence, the primer design for MCDA assay is a disadvantage for its more widespread application, when targeting sequences containing complex secondary structure, shorter specific regions or highly polymorphic markers. For this case, S-MCDA technology may be another option.

As an alternative to MCDA assay, the S-MCDA also achieves highly analytical specificity and this is due to the fact that a set of 7 primers for S-MCDA spanning 8 distinct regions of the target sequence was designed. The analytical sensitivity of *L. monocytogenes* S-MCDA method was 125 fg genomic DNA per test and 80-fold more sensitive than PCR, and the specificity was 100%^13^,^15^. What’s more, the S-MCDA technology provides a wider range of options for primer design since several of those primers can be positioned with fewer restrictions. For the S_1_-MCDA (S_2_-MCDA) assay, R1, CP2 and F2 (R2, CP1 and F1) primers can be designed with fewer restrictions, and the binding sites were not orientated in several particular regions.

## Conclusions

A novel nucleic acid amplified detection method was established on the basis of isothermal strand-displacement polymerization reaction. The MCDA assay developed here was rapid, robust, specific and sensitive, which had the advantages over the conventional PCR technique, namely, modest equipment requirements, easy operation, quick results, cost and energy efficiency, and high sensitivity and specificity. Moreover, the novel technology eliminated the thermal cycle steps (an isothermal amplification at a single temperature), and incubating in a short time (40-min for MCDA, 50-min for S-MCDA) using a simple block heater or water bath was sufficient to replicate nucleic acids to detectable levels. Herein, these attractive traits will motivate the researchers to explore the application of the novel MCDA technique for nucleic acid analysis in various fields.

## Methods

### Design of the MCDA primers

**Figure 2:**
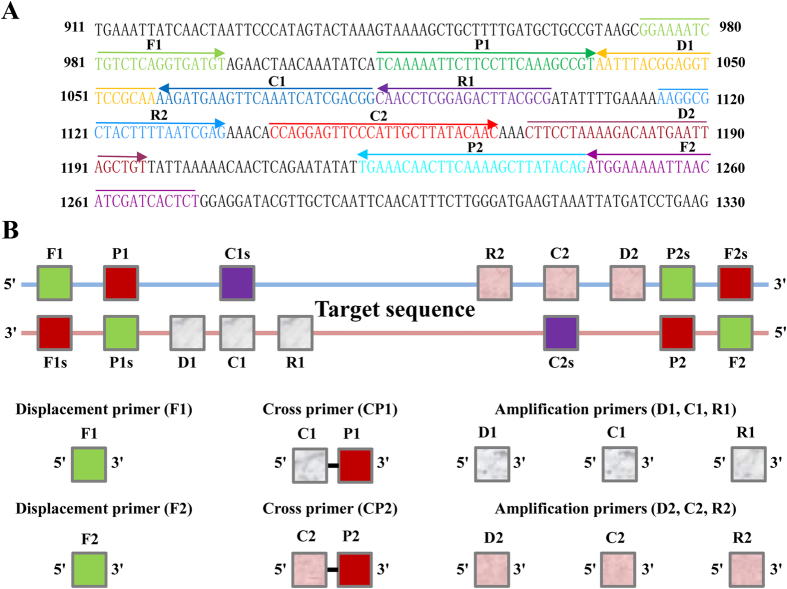
Schematic of primers’ sequence and positions for MCDA. (**A**) Location and nucleotide sequence of *L. monocytogenes hlyA* gene used to design the MCDA primers. The sequences of the primer sites are underlined. Right and left arrows indicate sense and complementary sequences that are used. (**B**) Schematic diagram showing the positions at which the primers attach to amplify the target sequence.

**Figure 3:**
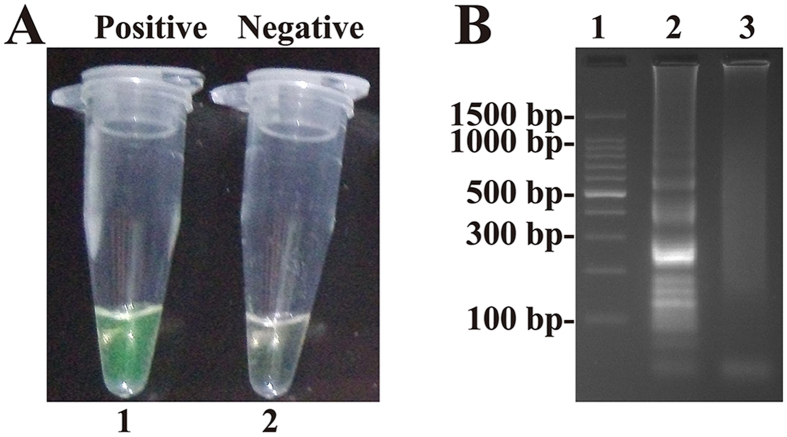
Confirmation and detection of MCDA products. (**A**) Color change of MCDA tubes; tube 1, positive amplification; tube 2, negative amplification. (**B**) 2.5% agarose gel electrophoresis applied to MCDA products; lane 1, DL 100-bp DNA marker; lane 2, positive MCDA products; lane 3, negative control (no DNA).

**Figure 4:**
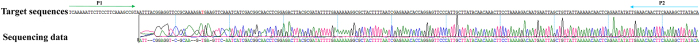
Sequencing analysis of MCDA amplified *L. monocytogenes hlyA* gene. The target sequeces were presented at the top and the sequences of the primers sites (P1 and P2) were underlined. Left and right arrows indicated complementary and sense sequences that were used. The sequencing data were shown in the bottom.

**Figure 5:**
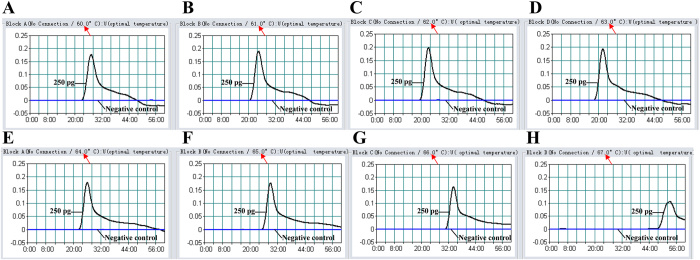
The optimal temperature for the MCDA assay. The MCDA reactions were monitored by real-time measurement of turbidity and the corresponding curves of concentrations of DNA were marked in the Figure. The threshold value was 0.1 and the turbidity of >0.1 was considered to be positive. Eight kinetic graphs (**A**–**H**) were obtained at different temperature (60–67 °C) with *L. monocytogenes* DNA at the level of 250 pg per reaction, and the graphs from B to F showed robust amplification.

**Figure 6:**
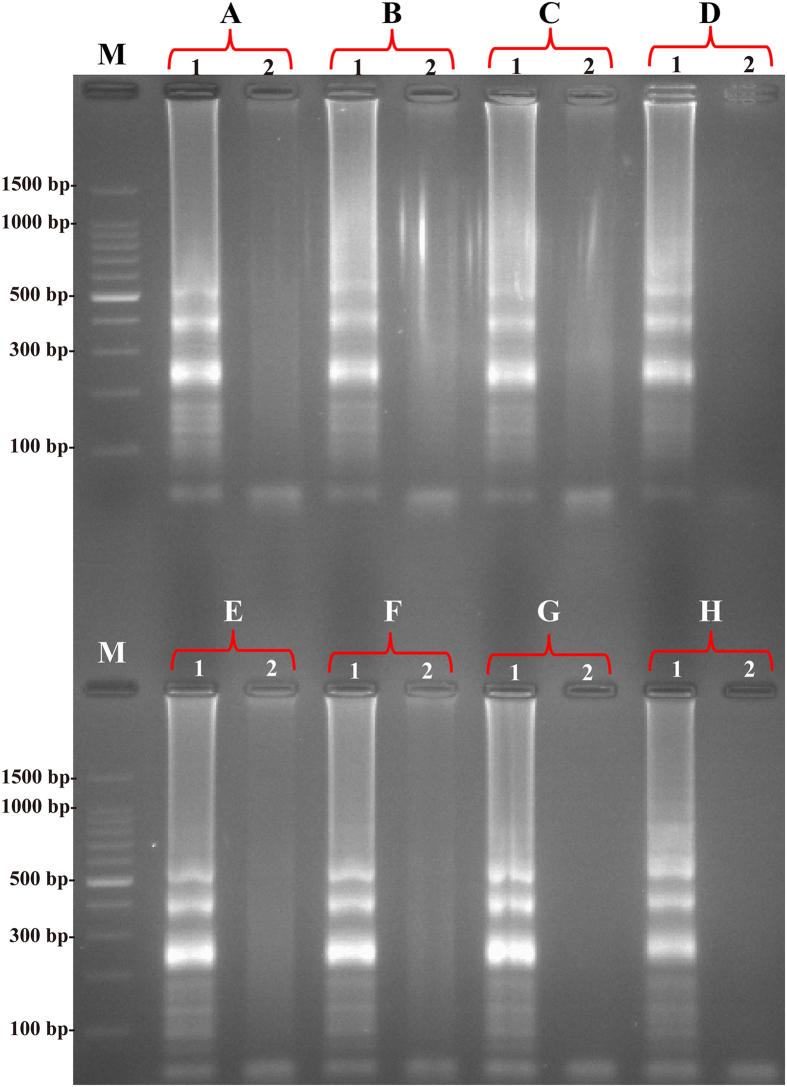
Products of MCDA monitored using 2.5% agarose gel electrophoresis. The products (**A**–**H**) of MCDA from different reaction temperature (60–67 °C) were monitor by 2.5% agarose gel electrophoresis after staining with ethidium bromide. Lane M, DL 100-bp DNA marker; lane 1, positive MCDA products; lane 2, negative control (no DNA).

**Figure 7:**
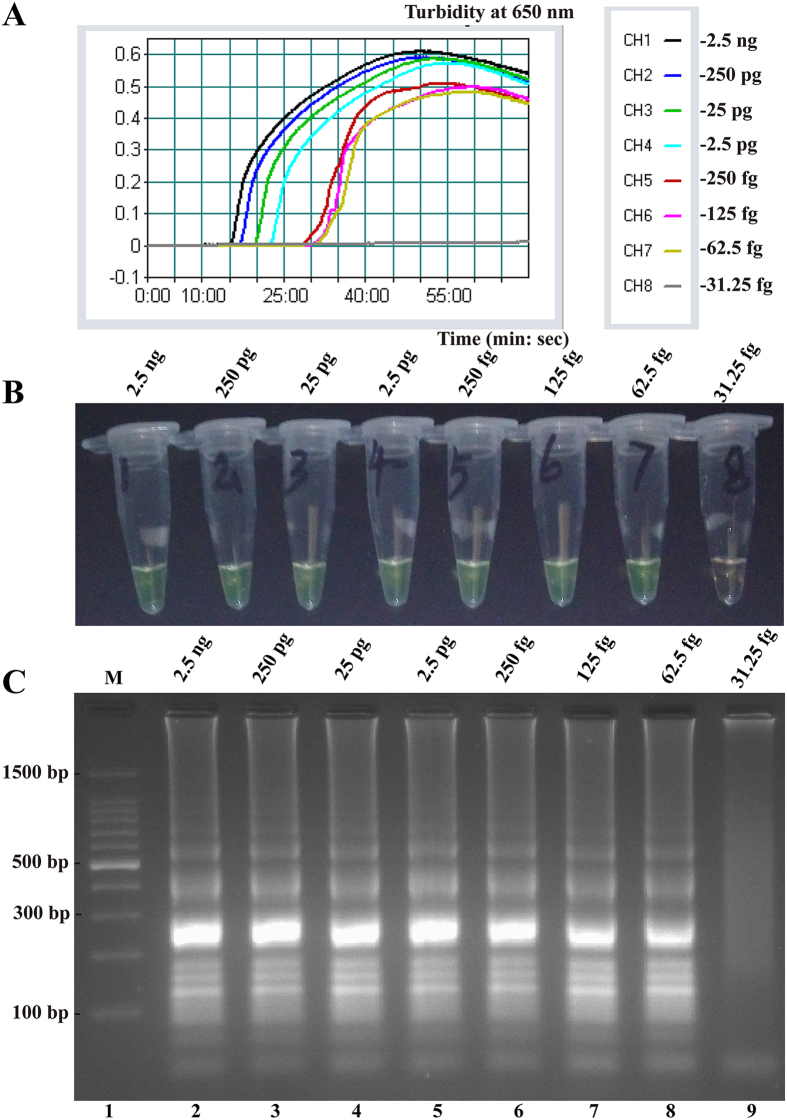
Sensitivity of the MCDA assays using serially diluted genomic DNA with *L. monocytogenes* strain EGD-e as template. (**A**) Sensitivity of MCDA for *L. monocytogenes* detection was monitored by real-time measurement of turbidity and the corresponding curves of concentrations of DNA were marked in the figure. The threshold value was 0.1 and the turbidity of >0.1 was considered to be positive. The LoD for MCDA approaches were 62.5 fg DNA per reaction and the MCDA reaction required only 40-min incubation periods at LoD levels of genomic DNA. (**B**) Sensitivity of MCDA methods for *L. monocytognenes* detection were visualized as color change by FD reagent. (**C**) Sensitivity of MCDA assays for *L. monocytognenes* detection were observed by 2.5% agarose gel electrophoresis, the positive products were observed as a ladder-like pattern on 2.5% agarose gel electrophoresis analysis. Lane 1, DL 100-bp DNA marker.

**Figure 8:**
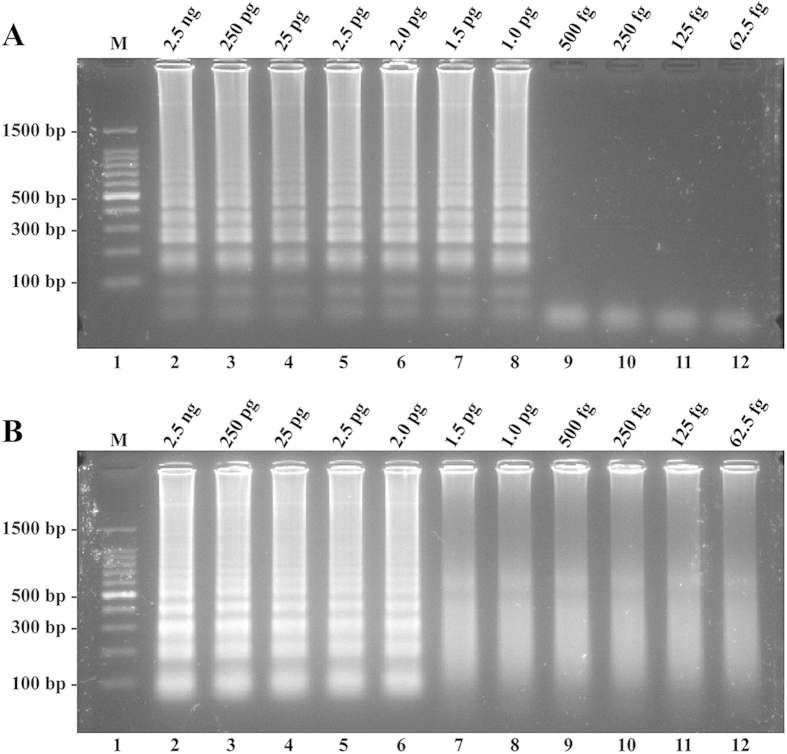
Sensitivity of the LAMP and CPA assays using serially diluted genomic DNA with *L. monocytogenes* strain EGD-e as template. Sensitivity of LAMP (**A**) and CPA (**B**) assays for *L. monocytognenes* detection were observed by 2.5% agarose gel electrophoresis, respectively. The positive products were observed as a ladder-like pattern on 2.5% agarose gel electrophoresis analysis. Lane 1, DL 100-bp DNA marker.

**Figure 9:**
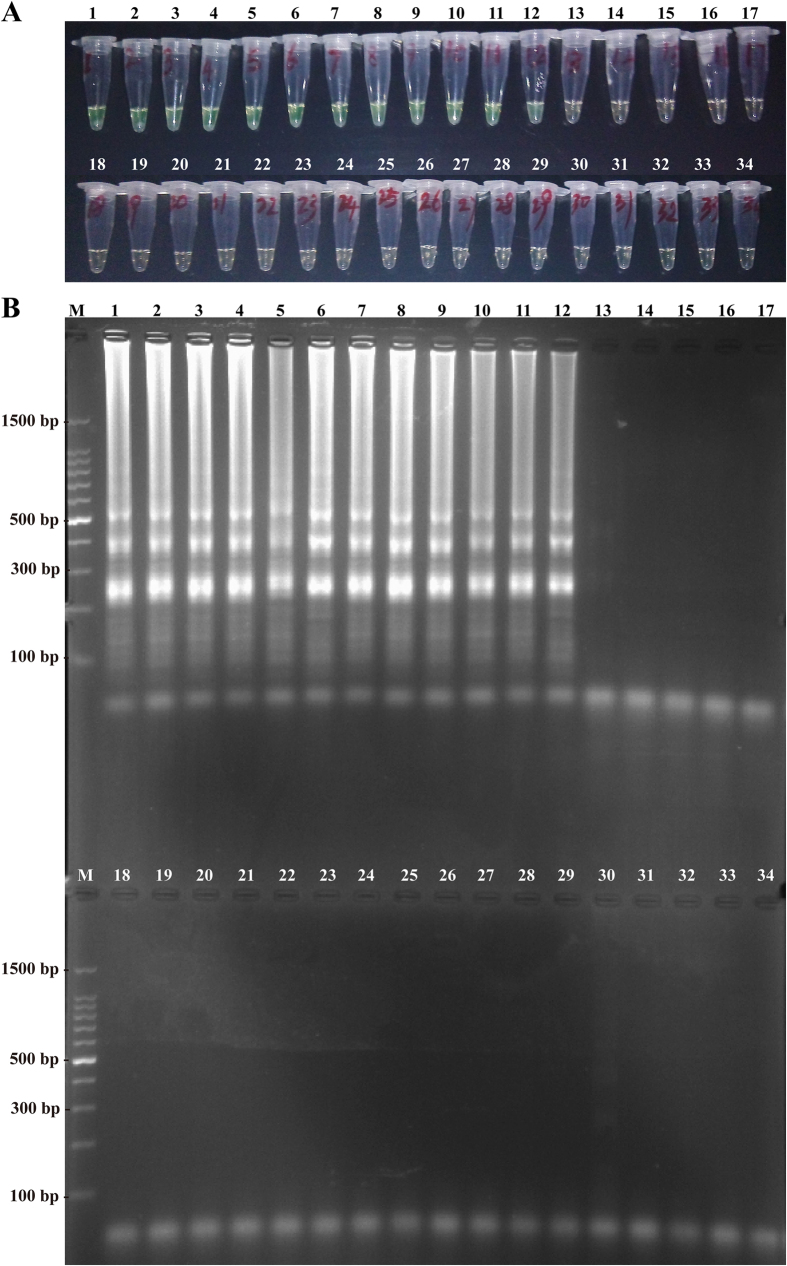
Specificity of MCDA detection for different strains. (**A**) The MCDA reactions were visualized as color change by FD reagent. (**B**) The MCDA products were observed as ladder-like pattern by 2.5% agarose gel electrophoresis. Tube (Lane) 1–12, *L. monocytogenes* strains of serovar 1/2a (EGD-e), 1/2b (ICDCLM007), 1/2c (ICDCLM010), 3a (ICDCLM023), 3b (ICDCLM078), 3c (ICDCLM446), 7 (NCTC10890), 4a (ATCC19114), 4b (ICDC419), 4c (ATCC19116), 4d (ATCC19117) and 4e (ATCC19118); tube (lane) 13-17, others *Listeria* reference strains of *L. innocua* (ATCCBAA-680), *L. ivanovii* (ATCCBAA-678), *L. seeligeri* (ATCC35967), *L. welshimeri* (ATCC35897), *L. grayi* (ATCC25402); tube (lane) 18-34, non-*Listeria* strains of *Enteropathogenic E. coli*, *Enterotoxigenic E. coli*, *Enteroaggregative E. coli*, *Enteroinvasive E. coli*, *Enterohemorrhagic E. coli*, *Enterobacter cloacae*, *Enterococcus faecalis*, *Bacillus cereus*, *Vibrio vulnificus*, *Vibrio fluvialis*, *Vibrio parahaemolyticus*, *Yersinia enterocolitica*, *Streptococcus pneumonia*, *Shigella flexneri*, *Plesiomonas shigelloides*, *Salmonella enteric*, *Klebsiella pneumonia*. Lane M, DL 100-bp DNA marker.

**Figure 10:**
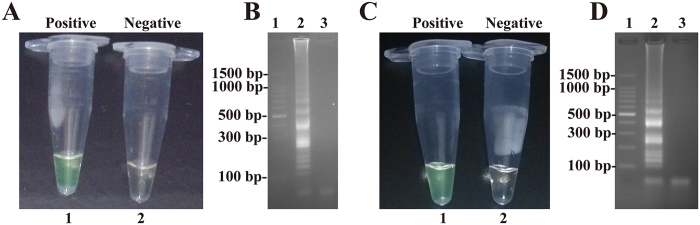
Confirmation and detection of S-MCDA products. (**A**,**C**): Color change of S_1_-MCDA (S_2_-MCDA) tubes; tube 1, positive amplification; tube 2, negative amplification. (**B**,**D**): 2.5% agarose gel electrophoresis applied to S_1_-MCDA (S_2_-MCDA) products; lane 1, DL 100-bp DNA marker; lane 2, positive MCDA products; lane 3, negative control (no DNA).

**Figure 11:**
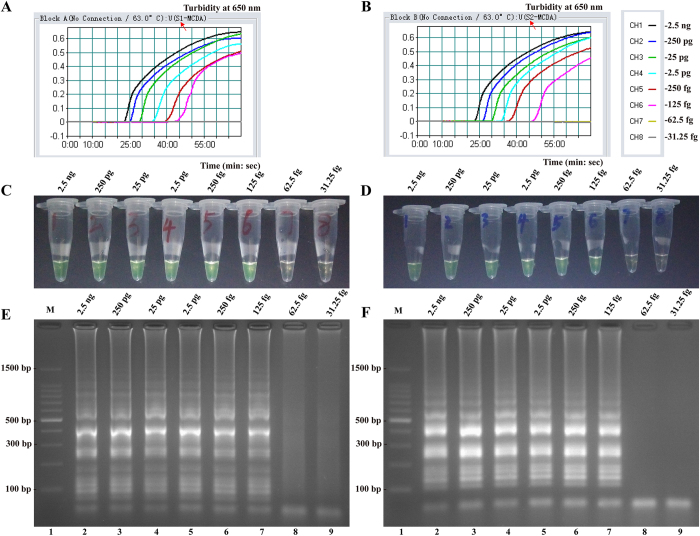
Sensitivity of the MCDA assays using serially diluted genomic DNA with *L. monocytogenes* strain EGD-e as template. (**A**,**B**) Sensitivity of S_1_-MCDA (S_2_-MCDA) for *L. monocytogenes* detection was monitored by real-time measurement of turbidity and the corresponding curves of concentrations of DNA were marked in the figure. The threshold value was 0.1 and the turbidity of >0.1 was considered to be positive. The LoD for S_1_-MCDA (S_2_-MCDA) assays were 125 fg DNA per reaction and the S-MCDA reaction required only 50-min incubation periods at LoD levels of genomic DNA. (**C**,D) Sensitivity of S_1_-MCDA (S_2_-MCDA) approaches for *L. monocytognenes* detection was visualized as color change by FD reagent. (**E**,**F**) Sensitivity of S_1_-MCDA (S_2_-MCDA) assays for *L. monocytognenes* detection was observed by 2.5% agarose gel electrophoresis, the positive products were observed as a ladder-like pattern on 2.5% agarose gel electrophoresis analysis. Lane M, DL 100-bp DNA marker.

*Listeria monocytogenes* (*L. monocytogenes*) was chosen as a model target microorganism for demonstrating the availability of MCDA assay. For the *L. monocytogenes*-specific *hlyA* gene, a set of 10 primers, which targeted 10 distinct regions, was designed based on the mechanism of MCDA by using PrimerExplorer V4 (Eiken Chemical) and primer software PRIMER PREMIER 5.0. The locations and sequences of MCDA primers were shown in Fig. 2 and Table 1.

### Materials

All of the oligomers were commercially synthesized and purified by Tsingke (Beijing, China). The Loopamp DNA amplification kits and Loopamp™ Fluorescent Detection Reagent (FD) were purchased from Eiken Chemical Co. Ltd. (Beijing, China). The DNA Extraction Kits (QIAamp DNA minikits) and QIAquick Gel Extraction Kit (QIAGEN, Hidlen, Germany) were purchased from Qiagen (Beijing, China).

### Bacterial strains

A total of 90 bacterial strains, including 40 non-*L. monocytogenes* and 50 *L. monocytogenes* strains, were used for the exclusivity and inclusivity tests, respectively (Table 3). All *Listeria* and other non-*Listeria* strains were cultured on brain heart infusion plates overnight at 37 °C. Strain EGD-e was used as a positive control to test the appropriate conditions for MCDA and to establish baseline specificity and sensitivity.

### Genomic DNA extraction

Bacterial genomic DNA was extracted from all cultured strains using DNA extraction kits (QIAamp DNA minikits; Qiagen, Hilden, Germany) according to the manufacturer’s instructions. DNA samples were stored at −20 °C.

### The MCDA assay

The MCDA reaction was performed with the Loopamp DNA amplification Kit in a final volume of 25 μl containing 2.4 μM each of cross primers CP1 and CP2, 0.4 μM each of displacement primers F1 and F2, 1.2 μM each of amplification primers R1, R2, D1 and D2, 0.8 μM each of amplification primers C1 and C2, 12.5 μl 2× reaction mix, 1 μl FD, 1.25 μl of *Bst* DNA polymerase (10 U) and 1 μl DNA template.

The MCDA amplification system can be separated into two independent single MCDA (S-MCDA) reactions, which are named as S_1_-MCDA and S_2_-MCDA. The primers set of S_1_-MCDA reaction system consists of CP1, CP2, F1, F2, R1, C1 and D1, S_2_-MCDA for CP1, CP2, F1, F2, R2, C2 and D2.

The S-MCDA reaction was also carried out in a total 25-μl reaction mixture containing 2.4 μM each of cross primers CP1 and CP2, 0.4 μM each of displacement primers F1 and F2, 1.2 μM each of amplification primers R1(R2) and D1(D2), 0.8 μM amplification primer C1 (C2), 12.5 μl 2× reaction mix, 1 μl FD, 1.25 μl of *Bst* DNA polymerase (10 U) and 1 μl DNA template.

Three mainstream methods were used to monitor the MCDA amplification. The color change of positive MCDA reactions could be directly observed by FD reagent, and the amplified products were also detected by electrophoresis on 2.5% agarose gels with ethidium bromide staining. Furthermore, real-time measurement of MCDA reactions was performed by recording the optical density (OD) at 650 nm every 6 s using the Loopamp Real-time Turbidimeter LA-320C (Eiken Chemical Co., Ltd, Japan). A positive amplification was defined as a threshold value of >0.1 and analysis of each sample (dilution) was tested at least two times.

In order to determine the optimal amplification temperature, the reaction mixtures of MCDA were incubated at a constant temperature ranging from 60 °C to 67 °C for 1 h and then heated at 95 °C for 5 min to stop the reaction. Mixture without DNA template was used as a negative control.

### Validation by sequencing

To further confirm the reliability and specificity of the MCDA assay, a MCDA-PCR-sequencing strategy was applied to analyze the MCDA products. The MCDA products corresponding to the level of 62.5 fg template DNA were verified by agarose gel electrophoresis, and the ladders between 100 bps and 1500 bps were extracted and purified by using QIAquick Gel Extraction Kit (QIAGEN, Hidlen, Germany) to obtain the template DNA for PCR. A set of two primers (P1 and P2) was used (Table 1), and PCR was performed in a final volume of 20 μl containing 10 mM Tris-HCl (pH 8.3), 50 mM KCl, 1.5 mM MgCl_2_, 0.001% gelatin, 0.2 μM each of P1 and P2 primers, 0.2 mM each of dNTPs, 0.5 μl DNA template, and 0.5 units of Taq DNA polymerase (ExTaq; Takara). The program consisted of the initial denaturation of 5 min at 95 °C, 32 cycles of 30 s at 95 °C, 30 s at 58 °C and 1 min at 72 °C, plus a final 5 min extension at 72 °C. The PCR products also were verified by electrophoresis, and then extracted and purified by using QIAquick Gel Extraction Kit (QIAGEN) again to obtain the template for sequencing. Sequencing was performed by Tsingke (Beijing, China) and the sequence data were compared with the target gene sequences in GenBank database.

### Analytical sensitivity of the MCDA assays

To test the limit of detection (LoD) of MCDA and S-MCDA approaches, amplification reactions were performed using serial dilutions (2.5 ng, 250 pg, 25 pg, 2.5 pg, 250 fg, 125 fg, 62.5 fg and 31.25 fg per microliter) of pure *L. monocytogenes* genomic DNA (strain EGD-e). 1 μl DNA template was added into the reaction mixtures and three replicates of each dilution were examined to assess the sensitivity of MCDA and S-MCDA assays.

To compare the sensitivity of the MCDA, LAMP and CPA assays in pure culture, template DNA from strain EGD-e was serially diluted (2.5 ng, 250 pg, 25 pg, 2.5 pg, 2 pg, 1.5 pg, 1 pg, 500 fg 250 fg 125 fg per microliter), which were used for accurately determining the LoD of LAMP and CPA approaches. The LoD of LAMP and CPA technologies were ascertained by genomic DNA amount of the template. The *hlyA*-LAMP method has been developed by Wang L *et al.* and the *L. monocytogenes*-CPA assay has been also established by Wang Y *et al.*, which were employed to confirm the LoD of LAMP and CPA methodologies^13^,^15^.

### Analytical specificity of the MCDA assays

To evaluate the specificity of the MCDA approaches, the MCDA reactions were carried out under the optimal conditions tested above with genomic DNA templates from the 40 non *L. monocytogenes* and 50 *L. monocytogenes* strains (Table 3). Analysis of each sample was tested at least two times.

### Practical application of MCDA to *L. monocytogenes* detection in food samples

To assess the practical feasibility of the novel MCDA method, we employed the *L. monocytogenes*-MCDA assay to the routine detection for 55 pork samples, and compared the detection results with the conventional PCR and ISO 11290-1 standard methods. A PCR assay, which was developed by Wang Y *et al.*, was carried out to verify the presence of *L. monocytogenes* strains in food samples^13^. The culture-biotechnical detection of *L. monocytogenes* was performed according to the manufacturer’s instructions of ISO 11290-1 method. In brief, 25 g of each pork sample was added into 225 ml *Listeria* enrichment broth (Half Fraser’s broth, Oxoid, Hampshire, UK), homogenized and incubated at 30 °C for 24 h. Then, 0.1 ml of each sample was placed into 10 ml of Fraser’s broth (FB) in a culture tube and incubated at 37 °C with shaking (250 rpm) for 48 h. Aliquots (0.05 ml) of positive FB cultures were plated on PALCAM agar (Oxoid), followed by incubating at 37 °C for 24 or 48 h. The identification of *L. monocytogenes* strains was confirmed by subsequent morphological, biotechnical and serological tests. Aliquots (1 ml) of the enriched cultures were subjected to extracted DNA, which was used as the template in the PCR and MCDA assays.

## Additional Information

**How to cite this article**: Wang, Y. *et al.* Rapid and Sensitive Isothermal Detection of Nucleic-acid Sequence by Multiple Cross Displacement Amplification. *Sci. Rep.* **5**, 11902; doi: 10.1038/srep11902 (2015).

## Figures and Tables

**Table 1:** Primers used for multiple cross displacement amplification.

**Primers**	**Sequence (5'-3')**	**Length**
CP1	CCGTCGATGATTTGAACTTCATCTT-TCAAAAATTCTTCCTTCAAAGCCGT	50 mer
CP2	CCAGGAGTTCCCATTGCTTATACAAC-CTGTATAAGCTTTTGAAGTTGTTTCA	52 mer
F1	GGAAAATCTGTCTCAGGTGATGT	23 nt
F2	AGAGTGATCGATGTTAATTTTTCCAT	26 nt
C1	CCGTCGATGATTTGAACTTCATCTT	25 nt
D1	TTGCGGAACCTCCGTAAATT	20 nt
R1	CGCGTAAGTCTCCGAGGTTG	20 nt
C2	CCAGGAGTTCCCATTGCTTATACAAC	26 nt
D2	CTTCCTAAAAGACAATGAATTAGCTG	26 nt
R2	AAGGCGCTACTTTTAATCGAG	21 nt
P1	TCAAAAATTCTTCCTTCAAAGCCGT	25 nt
P2	CTGTATAAGCTTTTGAAGTTGTTTCA	26 nt

**Table 2:** Limit of detection and time for MCDA method targeting *L. monocytogenes*, compared to LAMP and CPA approaches.

**Assays**	**Regions recognized**	LoD (no./reaction)*	**Fastest time (min)**	**Time for LoD (min)**
MCDA	10	62.5 fg	15	40
LAMP	8	1 pg	25	52
CPA	5	2 pg	33	63

^*^LoD values were the lowest DNA level that was positively detected in triplicate.

**Table 3:** Bacterial strains used in this study.

**Bacteria**	**Serovar**^a^	**Strain name.(source of strain)**^b^	**No. of strains**
*Listeria monocytogenes*	1/2a	EGD-e	1
		Isolated strains (ICDC)	6
	1/2b	Isolated strains (ICDC)	6
	1/2c	Isolated strains (ICDC)	6
	3a	Isolated strains (ICDC)	5
	3b	Isolated strains (ICDC)	1
	3c	Isolated strains (ICDC)	1
	7	NCTC10890	1
	4a	ATCC19114	1
		Isolated strains (ICDC)	5
	4b	Isolated strains (ICDC)	6
	4c	ATCC19116	1
		Isolated strains (ICDC)	2
	4d	ATCC19117	1
		Isolated strains (ICDC)	5
	4e	ATCC19118	1
		Isolated strains (ICDC)	1
*Listera ivanovii*	U	ATCCBAA-678	1
	U	Isolated strains (ICDC)	5
*Listera innocua*	U	ATCCBAA-680	1
	U	Isolated strains (ICDC)	2
*Listeria grayi*	U	ATCC25402	1
	U	Isolated strains (ICDC)	2
*Listeria seeligeri*	U	ATCC35967	1
	U	Isolated strains (ICDC)	1
*Listeia welshimeri*	U	ATCC35897	1
	U	Isolated strains (ICDC)	3
*Bacillus cereus*	U	Isolated strains (ICDC)	2
*Enteropathogenic E. coli*	U	Isolated strains (ICDC)	1
*Enterotoxigenic E. coli*	U	Isolated strains (ICDC)	1
*Enteroaggregative E. coli*	U	Isolated strains (ICDC)	1
*Enteroinvasive E. coli*	U	Isolated strains (ICDC)	1
*Enterohemorrhagic E. coli*	U	EDL933	1
*Plesiomonas shigelloides*	U	ATCC51903	1
*Shigella flexneri*	U	Isolated strains (ICDC)	2
*Enterobacter cloacae*	U	Isolated strains (ICDC)	1
*Enterococcus faecalis*	U	ATCC35667	1
*Yersinia enterocolitica*	U	ATCC23715	1
*Streptococcus pneumoniae*	U	Isolated strains (ICDC)	1
*Aeromonas hydrophila*	U	ATCC7966	1
*Vibrio vulnificus*	U	Isolated strains (ICDC)	1
*Proteus vulgaris*	U	Isolated strains (ICDC)	1
*Vibrio fluvialis*	U	Isolated strains (ICDC)	1
*Streptococcus bovis*	U	Isolated strains (ICDC)	1
*Vibrio parahaemolyticus*	U	ATCC17802	1
*Klebsiella pneumoniae*	U	ATCC700603	1
*Salmonella enteric*	U	ATCC14028	1

^a^U, unidentified serotype.

^b^ATCC, American Type Culture Collection; NCTC, National Collection of Type Cultures; ICDC, National Institute for Communicable Disease Control and Prevention, Chinese Center for Disease Control and Prevention.

**Table 4:** Comparison of conventional PCR, MCDA and culture-biotechnical method, for the detection of *L. monocytogenes* in pork samples.

**Detection approaches**	**Pork samples (n = 55)**	
	**Positive**	**Negative**
PCR	8	47
MCDA	14	41
Culture	14	41
